# Value of ^18^F FDG-PET/CT parameters on long term follow-up for patients with non-small cell lung cancer

**DOI:** 10.1515/iss-2022-0009

**Published:** 2022-10-05

**Authors:** Mohammed Zoair, Samantha Taber, Roland Bittner, Gregor Foerster, Sergej Griff, Torsten T. Bauer, Joachim Pfannschmidt

**Affiliations:** Department of Thoracic Surgery, Heckeshorn Lung Clinic, HELIOS Klinikum Emil von Behring, Berlin, Germany; Department of Radiology and Nuclear Medicine, HELIOS Klinikum Emil von Behring, Berlin, Germany; Institute of Pathology, HELIOS Klinikum Emil von Behring, Berlin, Germany; Department of Pneumology, Heckeshorn Lung Clinic, HELIOS Klinikum Emil von Behring, Berlin, Germany

**Keywords:** lung cancer, PET/CT, staging, surgery, survival

## Abstract

**Objectives:**

The purpose of this study was to investigate the value of PET/CT in the preoperative staging of non-small cell lung cancer in predicting long-term survival and diagnostic performance, validated by histopathology following surgical resection.

**Methods:**

Between 02/2009 and 08/2011, 255 patients with non-small cell lung cancer were included in this single-center prospective study. All underwent 18F FDG-PET/CT for pre-operative staging, and in 243 patients complete surgical resection was possible. Regarding lymph node involvement and extrathoracic metastases, sensitivity, specificity, positive predictive value (PPV), and negative predictive value (NPV) were calculated using the histopathological staging as reference. Median follow-up for censored patients was 9.1 years.

**Results:**

Overall 5-year survival rate of all patients was 55.6%, and of patients who had complete surgical resection it was 58.2%. In multivariate analysis of all surgically resected patients lymph node involvement (p=0.029) and age >61 years (p=<0.001) were significant independent prognostic factors. SUVmax and SUVmean cut-offs between SUV 2 and 11, however, were not associated with better or ;worse survival. The PET-CT sensitivity, specificity, positive predictive value and negative predictive value for predicting lymph node involvement were 57, 95, 88, and 76%, respectively. Furthermore, sensitivity, specificity, positive predictive value, and negative predictive value for detecting extrathoracic metastases were 100, 58, 98, and 100%, respectively.

**Conclusions:**

In this study, tumor 18F FDG-uptake values did not provide additional prognostic information. Age>61 years and lymph node metastasis were associated with worse long-term survival in surgically resected patients. 18F FDG-PET/CT scans allow for improved patient selection. However, in staging mediastinal lymph nodes, there is a high rate of false positives and false negatives, suggesting that tissue biopsy is still indicated in many cases.

## Introduction

Lung cancer is the leading cause of cancer-related death in Germany and worldwide [1]. Staging and classification are important for optimizing management, estimating prognosis, and evaluating treatment results. In non-small cell lung cancer, CT with 18-fluorodeoxyglucose positron emission tomography (^18^F FDG-PET/CT) has been recommended as the standard for establishing TNM disease stage. It is a hybrid imaging modality that combines radiomorphologic-anatomic information with metabolic information, and is considered an important tool for estimating malignancy in primary tumors and detecting thoracic lymph node metastases or extrathoracic metastases. In this prospective study, we investigate whether the use of ^18^F FDG-PET in association with CT provides better preoperative staging for NSCLC and whether it can help predict long-term survival over a long time frame, independently of tumor stage.

## Patient and methods

Beginning in 02/2009 and continuing through 08/2011, 255 NSCLC patients were recruited from the Heckeshorn Lung Clinic. Patients with histologically established non-small cell lung cancer were eligible to participate in this prospective study. Patients were excluded if they were less than 18 years of age, had a history of type I diabetes, ongoing drug abuse, inability to give informed consent, were either pregnant or breast-feeding, or had had a known cancer within 2 years of the PET/CT. None of the patients had induction systemic or radiation therapy. All patients had to be able to tolerate surgical resection if determined technically and oncologically feasible. The local institutional review board (Berlin Aeztekammer Eth-15/09) approved the study.

Patients were evaluated preoperatively by physical examination, bronchoscopy, chest radiograph, and ^18^F FDG-PET/CT-scans. Brain-CT or magnetic resonance imaging scans were performed when clinical signs and symptoms suggestive of cerebral metastases were present. In patients with advanced stage lung cancer MRI was routinely performed. However, in patients with stage I and II disease, cerebral staging relied mainly on the contrast-enhanced ^18^F-FDG PET/CT imaging protocols.

After surgery patients were followed for up to five years. In the first two years a chest CT and an ultrasound or CT of the abdomen were performed every six months. For three further years, follow-ups were performed every 12 months. After this time, examinations were only performed when symptoms suspicious for a recurrence were present. All patients were preoperatively and postoperatively staged according to the TNM classification system UICC 7 [2] and reclassified according to UICC 8 [3]. Preoperative staging was based on ^18^F FDG-PET -scans, which were interpreted by a board-certified nuclear radiologist (G.F.), and on CT scans, which were interpreted by a chest radiologist (R.B.). Sensitivity, specificity, positive predictive value, and negative predicted value were determined for ^18^F FDG-PET/CT, using the pathology results of surgical resection as the reference.

Although all patients had bronchoscopy prior surgery, invasive lymph node staging by EBUS-bronchoscopy or mediastinoscopy was primarily performed in patients with suspected mediastinal lymphadenopathy. Patients suspected of having mediastinal disease in a location inaccessible by EBUS bronchoscopy or mediastinoscopy underwent thoracoscopy, and/or thoracotomy to determine nodal stage. Patients with suspected M1 disease of the liver, adrenal glands, or contralateral lung underwent definitive biopsy to determine whether metastases were present. If the bone or brain was suspected to harbor metastases, magnetic resonance imaging was considered the standard reference. Patients with established N3 or M1 disease were included in the documentation, but surgery was not performed. Patients with clinically staged localized, non-bulky N2 disease or lower had primary pulmonary resection. All surgeries were performed in curative intent and included lobectomy, pneumonectomy, or sublobar resection. All pulmonary resections also included systematic hilar and mediastinal lymph node dissection, performed according to standard practice [4]. Thus, right-sided lung resections included dissection of the paratracheal, subcarinal, inferior mediastinal, interlobar, and hilar lymph nodes, and left-sided resections included dissection of aortic, infracarinal, inferior mediastinal, interlobar, and hilar lymph nodes. Pathologic review was performed using standard techniques and complemented with immunohistochemical staining. Pathologic specimens were assessed according to the IASLC map for patterns of tumor spread, and lobar (stations 12 and 13), hilar (station 10), and interlobar (station 11) lymph node metastases were classified as N1. In patients with acceptable ECOG performance status and pathologically proven N1 involvement adjuvant chemotherapy was administered. Patients with pN2 disease were treated with postoperative radiation of the mediastinum as well.

### ^18^F FDG-PET/CT imaging protocol

Imaging was performed using an integrated PET/CT system (Siemens Biograph16, München, Germany). All patients had fasted for at least 6 h and had confirmed normal blood glucose levels prior to the intravenous injection of 250 MBq of 18F-FDG. After a 30-min phase of resting, all patients received an oral, diluted contrast agent (12.5 mL Peritrast Oral CT 400 mg Iod/ml in 500 mL water; Dr. F. Köhler Chemie GmbH, Bensheim, Germany). After an additional 30 min, the intravenous contrast agent was injected, and then after a delay of 70 s the CT scan was performed from the base of the skull to the upper thighs (120 kV, 150 mAs (Care Dose 4D) and 2-mm section thickness). Immediately after helical CT scanning, a PET emission scan that covered the identical transverse field of view was obtained with 3-min acquisition time per bed position. Finally, the data were reconstructed using the CT scan for attenuation correction. Image interpretation involved both qualitative (visual) and semi-quantitative analysis of the lesions using the mean standardized uptake value (SUV) of FDG within a 50%-threshold volume of the maximum-pixel SUV and the maximum-pixel SUV itself (SUVmean/max). Lesions with SUVs of ≥2.5 were considered PET positive and indicative of pulmonary or extrapulmonary disease. SUVmax and SUVmean were also documented and considered in the statistical analysis.

### Statistical methods

Data was stored in an Access database (Microsoft, Seattle, WA) and analyzed with SPSS Statistics 22.0 (IBM Corp, Armonk, NY). The distribution of the categorical variables in the different groups was tested with Fisher’s exact test and the χ^2^test. The relationships between SUVmax and SUVmean of the primary NSCLC and the different variables were analysed using appropriate non-parametric statistic tests. For all cases, overall survival time was calculated as the time interval between lung resection and last follow-up date. For patients who died from lung cancer or other causes, the last follow-up date was defined as the date of death. Kaplan-Meier methods were used to estimate survival probabilities [5]. We used the log-rank test to analyze differences between subgroups, and we used Cox’s proportional hazards model to evaluate incremental risk factors influencing survival. Variables with a significance level of p<0.2 in the univariate survival analysis were included in the multivariate analysis. p-values of less than 0.05 were considered statistically significant. Sensitivity, specificity, positive predicted value, negative predicted value, and accuracy were determined for F-18-FDG-PET/CT, using the pathology results of surgical resection and/or biopsy as the reference.

## Results

### Patient characteristics and overall staging

The baseline characteristics for all 255 patients are shown in Table 1. The majority were male (63.5%) and had stage I or II lung cancer (73.7%). 45.9% of all cancers were adenocarcinomas, 41.6% were squamous cell carcinomas, 6.3% were partial neuroendocrine differentiated carcinomas, 4.7% were large cell carcinomas, and 1.6% were sarcomatoid differentiated.

**Table 1:** Patient characteristics.

Characteristics	Cases (%)
Median age (range)	66 years (40-87 years) mean 65 years (±9 years)
Gender	162♂ (63.5%), 93♀ (36.5%)
SUV max primary tumor (mean ± SD (range))	11.06 ± 6.2 (1.5–42.6)

Tumor pathological stage (T)	

T1	77 (30.2%)
T2	120 (46.3%)
T3	48 (18.8%)
T4	10 (3.9%)

Nodal pathological stage (N)	

N0	150 (58.8%)
N1	61 (23.9%)
N2	43 (16.9%)
N3	1 (0.4%)

Pathological stage (UICC8)	

I	96 (37.6%)
	IA1:29 (11.4%)
	IA2:28 (11.0%)
	IB:39 (15.3%)
II	93 (36.5%)
	IIA: 21 (8.2%)
	IIB: 72 (28.2%)
IIIa/IIIb	59 (23.1%)
	IIIA: 46 (18.0%)
	IIIB:13 (5.1%)
IVa	7 (2.7%)

Procedure/surgery	

Lobectomy	152
Extended	26
Peumonectomy	33
Segmentectomy	20
Wedge resection	22
No resection	2
Mediastinoscopy or VAMLA	38
EBUS	5

Histology	

Adenocarcinoma	117 (45.4%)
Squamous cell carcinoma	104 (40.7%)
Neuroendocrine differentiated	15 (5.8%)
Large cell carcinoma	4 (1.6%)
Miscellanous histologies	10. (3.9%)

The most frequently performed surgeries were lobectomy or extended lobectomy (i.e., with bronchoplasty) (70.2%). In 243 patients (95.3%) complete surgical resection was possible. 92 patients (36.1%) received platin-based adjuvant chemotherapy, and 32 patients (12.5%) received adjuvant radiation therapy. The median duration between preoperative ^18^F-FDG PET/CT and attempted curative resection was 16 days.

Censored subjects were followed for a median time of 9.1 years (95% CI: 8.8–9.4 years). During this observation period 142 patients died, and the overall-5-year survival rate was 55.6%

**Figure 1:**
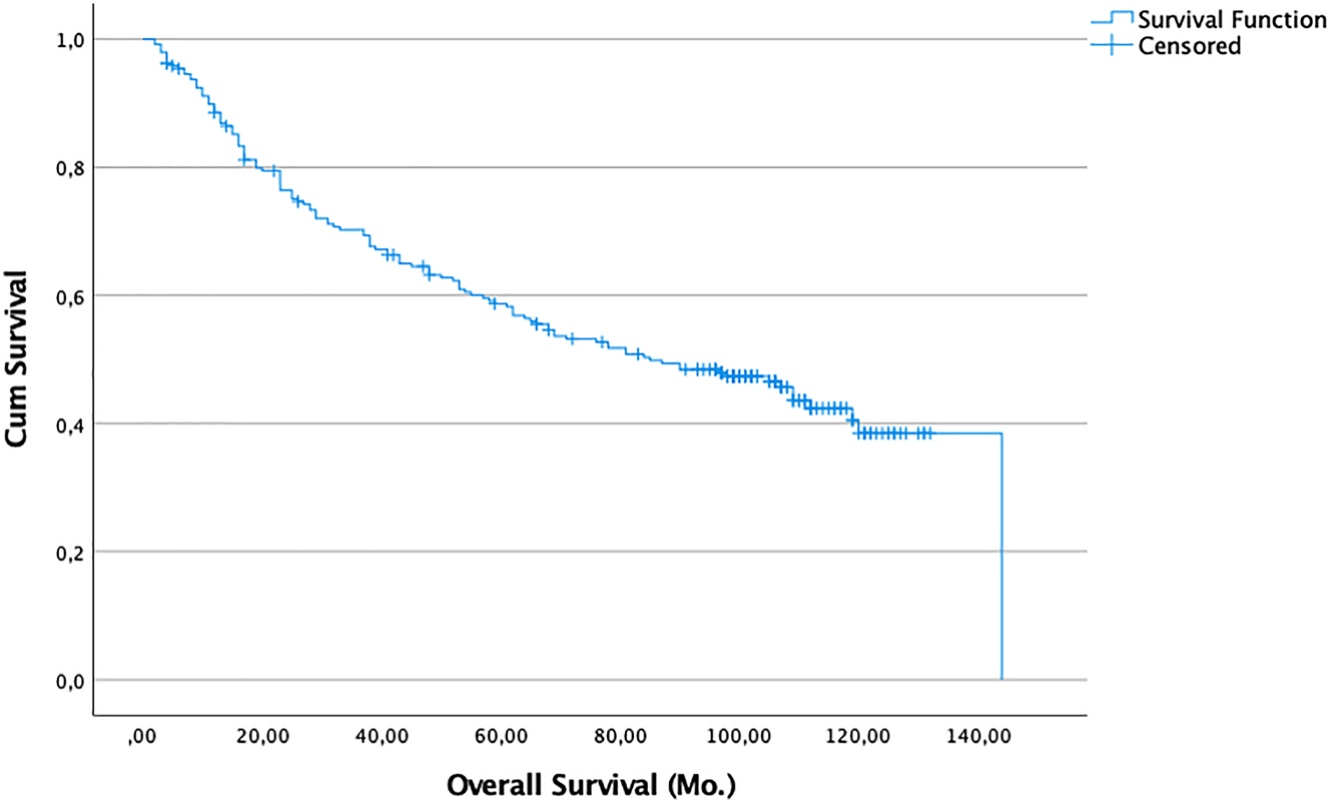
Probability of survival (death from any cause) of patients with R0 resection (n=238). Zero time on the abscisse represents the date of pulmonary resection.

We performed a post hoc analysis of curatively (R0) resected patients (n=238). For this analysis we excluded the 7 patients who had died within 30 days of surgery in order to eliminate the potentially confounding factor of death from other non-tumor related complications. Here, the 5-year overall survival rate was 58.2%, and the median survival was 85 months (95% CI: 61.4–108. 6 months) (Figure 1). At the end of the study period 111 of these patients had died, 10 of them from non-tumor related causes.

**Figure 2:**
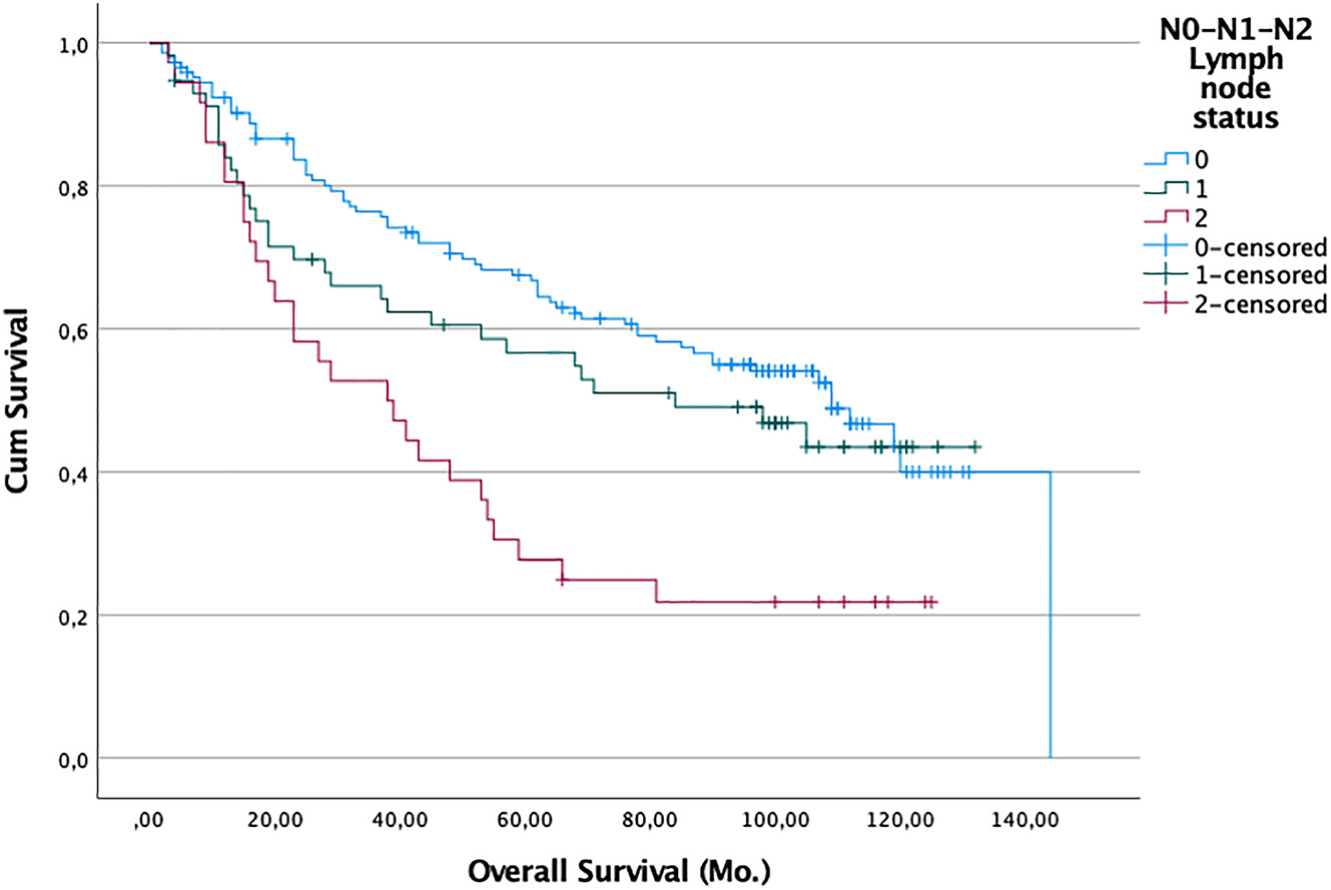
Probability of survival (death from any cause) of patients with R0 resection (n=238). Zero time on the abscisse represents the date of pulmonary resection. Difference in survival between all groups: Log-rank analysis p: <0.001. blue line: pN0 lymph node status; n=145 pat. green line: pN1 lymph node status; n=57 pat. violet line: pN2 lymph node status; n=36 pat.

**Figure 3:**
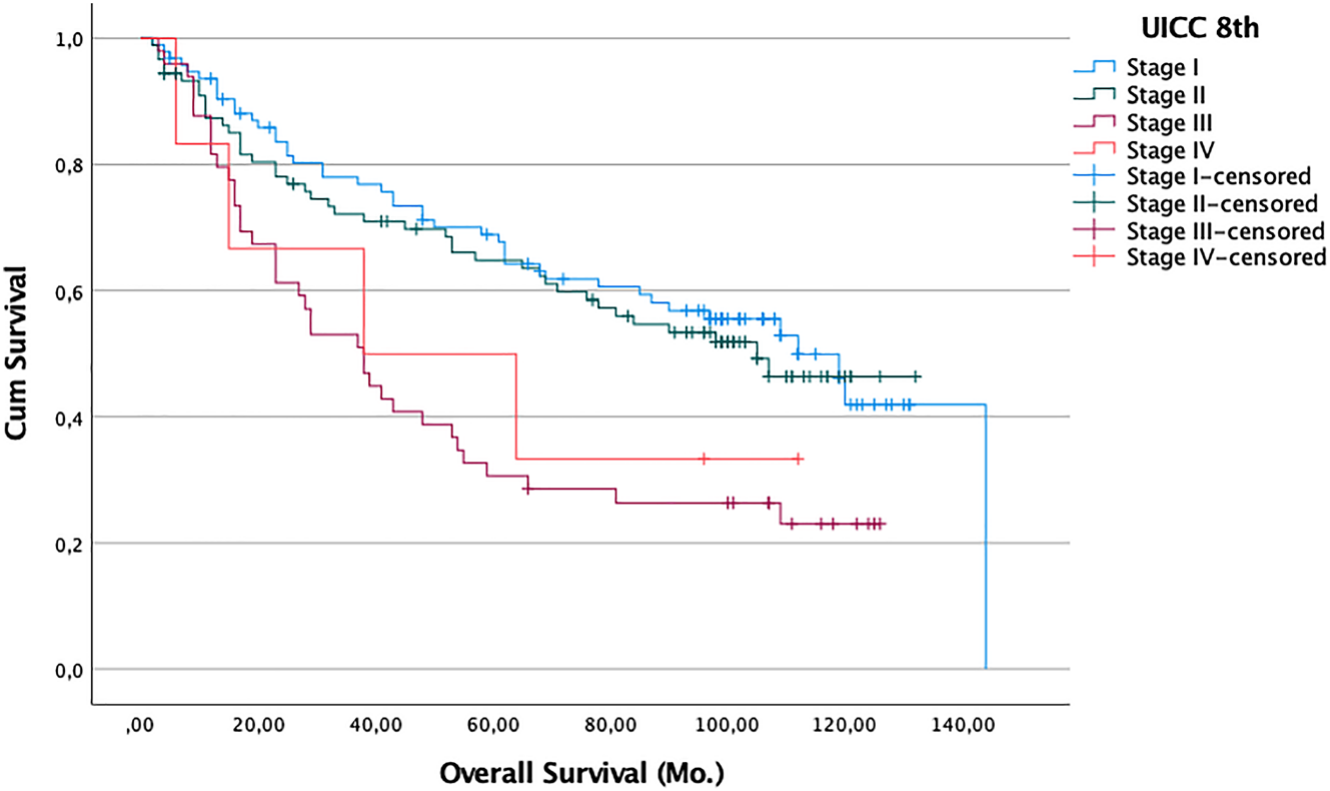
Probability of survival (death from any cause) of patients with R0 resection (n=238). Zero time on the abscisse represents the date of pulmonary resection. Difference in survival between all groups: Log-rank analysis p: <0.001. blue line: Stage I; n=94 pat. green line: Stage II; n=89 pat. violet line: Stage III; n=49 pat. red line: Stage IV; n=6 pat.

**Figure 4:**
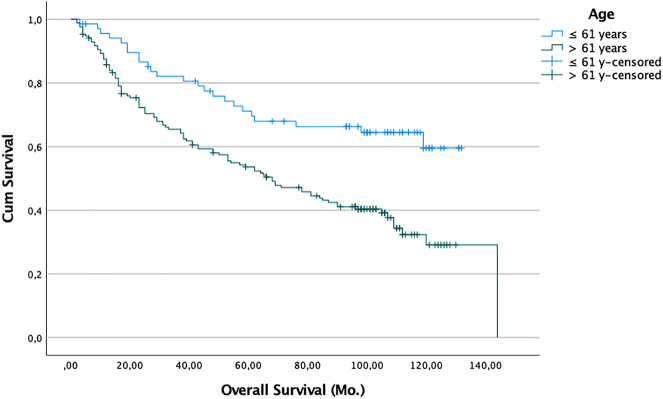
Probability of survival (death from any cause) of patients with R0 resection (n=238). Zero time on the abscisse represents the date of pulmonary resection. Difference in survival between groups: Log-rank analysis p: <0.001. blue line: age ≤61 years; n=69 pat. green line: age >61 years; n=169 pat.

We also performed sensitivity analyses to try to establish cut-offs values for SUVmax or SUVmean that might help predict which patients would survive long-term. We evaluated potential SUVmax and SUVmean cut-offs of between SUV 2 and 11 but found that even in the univariate analysis none of these were associated with better or worse chances of survival (p>0.05). Of the additional factors analyzed, sex, resection type, tumor size, and histology of the primary tumor had no significant influence on survival. However, higher pathological nodal stage (Figure 2), higher UICC 8 stage (Figure 3), and greater age (>61 vs. ≤61 years) (Figure 4) were associated with worse survival outcomes in the univariate analysis. Median survival for patients with pN0 (145 pat) was 109 months (95% CI: 84.7–133.2), for pN1 (57 pat) it was 84 months (95% CI: 35.7–132.3), and for pN2 (36 pat) it was 38 months (95% CI: 17.4–58.6) (p:<0.001). For patients with stage I (94 pat.) median survival was 112 months (95% CI: 91.8–132.2), for stage II (89 pat) 105.0 months (95% CI: n.def.), for stage III (49 pat) 38.0 months (95% CI: 6.4–50.5), and for stage IV (6 pat) 38.0 months (95% CI: 0.0–96.8) (p>.001). For patients <61 years (69 pat) the median survival time was not reached vs. age >61 (169 pat): 68 months (95% CI: 48.4–87.6) (p<0.001).

Our multivariate analysis of all surgically resected patients (n=238) included UICC 8 stage, lymph node involvement (N0 vs N1/N2), and age (≤61/>61 years). Lymph node involvement (p=0.029) and age >61 years (p=<0.001) retained significance as independent prognostic factors, whereas higher UICC 8 stage showed only a non-significant trend toward worse survival (p=0.136) (Table 2).

**Table 2:** Relationship of individual variables to death (Cox proportional hazard method).

Variable	p-Value	Hazard ratio	95% confidence limit
Lymph node involvement	0.029	1.4	(1.04–1.90)
UICC8	0.136	1.2	(0.934–1.65)
Age (≤61,/>61 years)	<0.001	2.6	(1.7–4.1)

### Rating the diagnostic value of ^18^F-FDG PET/CT

For primary tumors, the mean SUVmax was 11.1 (SD: ±6.2), and the mean SUVmean was 7.5 (SD: ±4.3). The sensitivity for ^18^F-FDG PET/CT scans (SUVmax ≥2.5) for detecting primary lung cancer was 96.8% (8 of 255 patients were PET negative); for pT1 tumors the sensitivity was 90.9% (7 of 77 patients were PET negative). The distribution of SUVmean and SUVmax of the primary tumors was not associated with the presence (or absence) of lymph node metastases (N1/N2). In the Kruskal-Wallis test, however, SUVmean and SUVmax were indeed associated with higher T categories: T1 tumors were significantly less PET-avid than tumors of higher T categories (p<0.001). We noted a high degree of variability in PET avidity between different lymph node stations, with a mean SUVmax of 8.3 (SD: ±4.9) in #2R and 0.8 (SD: ±1.6) in #3; the mean SUVmean was 5.9 (SD: ±3.7) in #2R and 0.6 (SD: ±1.1) in #3. The sensitivity, specificity, PPV, NPV, and accuracy for ^18^F-FDG PET/CT scans for detecting extrathoracic metastases and thoracic lymph node involvement (N1/N2) are presented in Table 3. As illustrated, ^18^F-FDG PET/CT has significantly higher sensitivity and specificity for the detection of extrathoracic metastases, compared to lymph node metastases. Of note, ^18^F-FDG PET/CT had a sensitivity of only 55% for predicting N2 subcarinal lymph node metastases (station #7). For the frequently dissected hilar lymph nodes in stations #10L and #10R the sensitivities were 55 and 80%, respectively. The sensitivity and specificity for each individual lymph node station are presented in Table 4.

**Table 3:** Diagnostic value of lymph node metastases and distant metastases by PET/CT.

Method	Lymph node involvement	Distant metastases (UICC:M1a/b/c)
Sensitivity	0.57 (95% CI: 0.47–0.67)	1.00 (95% CI: 0.59–1.00)
Specificity	0.95 (95% CI:0.90–0.98)	0.98 (95% CI: 0.95–0.99)
PPV	0.88 (95% CI: 0.78–0.95)	0.58 (95% CI: 0.28–0.85)
NPV	0.76 (95% CI: 0.69–0.82)	1.00 (95% CI: 0.99–1.0)

**Table 4:** Depicts the sensitivity, specificity, positive predictive value, and negative predictive value of PET/CT in different lymph node stations.

Lymph node station #	N	Sensitivity	Specificity	PPV	NPV
2L	0	n.d.	1.0	n.d.	1.0
2R	3	0.29	0.99	0.67	0.98
3anterior	3	0.00	0.98	0.0	0.99
3posterior	4	n.d.	0.99	0.0	1.0
4L	5	0.75	0.99	0.60	0.99
4R	22	0.65	0.96	0.59	0.97
5	8	0.83	0.98	0.62	0.99
6	1	1.00	1.00	1.00	1.00
7	32	0.55	0.97	0.71	0.95
8L	0	n.d.	1.00	n.d.	1.00
8R	1	0.33	1.00	1.00	0.99
9L	3	0.50	0.99	0.33	0.99
9R	0	0.00	1.00	n.d.	0.99
10L	14	0.55	0.98	0.71	0.96
10R	32	0.80	0.95	0.62	0.98
11L	0	0.00	1.00	n.d.	0.87
11R	5	0.11	1.00	1.00	0.84

^18^F-FDG PET/CT scan was highly sensitive and specific for determining the presence or absence of metastatic disease in 2 patients with adrenal gland metastases, 2 patients with hepatic metastases, and 3 patients with pleural metastases. Using the histologic findings as reference ^18^F-FDG PET/CT scans overestimated the T status in 15.7% of cases and the lymph node status in 18.8% of cases. In contrast, it suggested an erroneously low T status in 9.8% of cases and a falsely low nodal stage in 2.4% of cases (Table 5).

**Table 5:** Disparity of PET/CT and pathological tumor-node-metastasis staging.

	T	N	M
Unchanged	190 (74.5%)	188 (73.7%)	249 (97.6%)
Upstaged	40 (15.7%)	48 (18.8%)	0 (0.0%)
Downstaged	25 (9.8%)	19 (7.5%)	6 (2.4%)

## Discussion

International guidelines recommend ^18^F-FDG PET/CT for staging of NSCLC patients, who are being considered for curative local therapy. Several studies have attempted to determine the prognostic significance of preoperative PET, specifically by investigating the relationship between long term prognosis and the maximum and mean SUVs measured in the primary tumor [6], [7], [8].

Our study confirms the ominous prognostic significance of hilar and mediastinal lymph node metastases and age >61 years in the multivariable analyses of surgically resected patients. In an attempt to define the role of ^18^F-FDG PET/CT in surgically resected adenocarcinoma of the lung, Ventura L et al. [9] found that male sex and the higher tumor stages III and IV were associated with poor overall survival. This is in line with results of a study by Vesselle H et al. [10]: in a subgroup analysis of 103 surgically resected patients with non-small cell lung cancer only tumor stage retained significance in a multivariate analysis for overall survival. One of the strengths of our study was the thorough preoperative staging, along with surgically verified pathological staging.

Several different SUVmaxes have been reported as significant predictors of good vs. poor long-term survival, with cut-offs ranging between 5 [7, 11]and 20 [12]. In their retrospective analysis of 315 surgically treated NSCLC patients Cerfolio et al. [13] found that SUVmax >10 was associated with significantly worse disease-free survival in patients with stage IB and stage II. Their multivariable analysis also compared stages I/II with stages III/IV and found that stage III/IV may be associated with higher SUV.

Other studies, however – including ours – found that SUV of the primary tumor had no impact on prognosis [10, 14]. What can explain these divergent findings?

The heterogeneity in PET imaging thresholds may be related to technical differences in PET scanners and scanning protocols. Pre-diagnostic patient preparation or the administration of neo-adjuvant chemotherapy may also play a role. Additionally, the analysis of SUVmax semi-quantitatively reflects the most active part of the tumor but may not sufficiently describe the metabolic characteristics of the tumor in its entirety. Moreover, variation in Glut-1-transporter enzyme activity likely influences SUV uptake as well, although it does not necessarily reflect tumor aggressivity [15]. Moreover, PET studies are often limited by their retrospective nature. Differences in histology, methodology, and study design make comparisons between the different studies difficult. Moreover, the decision of which covariates to include in the final analysis plays a crucial role. In the above cited study by Sasaki et al. for example, although SUVmax >5 did associate with worse prognosis, tumor size, which was a strong prognostic factor in the univariate analysis, was not included in the multivariate analysis [7].

Based on the findings of our study and on the mixed findings from other studies, we conclude that– especially when tumor size and stage are taken into account – there is too little evidence to establish a clear link between FDG uptake in primary NSCLC and prognosis. Therefore, although ^18^F-FDG PET/CT continues to provide critical information for establishing preoperative tumor stage, clinicians should not be misled into letting metabolic activity of the primary tumor, as registered by ^18^F-FDG PET/CT, affect their treatment decisions – to forgo potentially curative surgery for example.

The management of T1 tumors (coin like solitary pulmonary nodules) in the routine staging process with ^18^F-FDG PET/CT is evolving. The reported sensitivity of 90.9% in our study for T1 tumors has also been shown in other publications, with reported sensitivities of up to 95 and 97% [16, 17]. However, the threshold of 8–10 mm in tumor size for PET examinations was set in international guidelines to underline the significant risk of false-negative findings for small coin lesions. To eliminate one of the main sources of false-negative and false-positive findings, PET should not be considered reliable for evaluating ground glass opacity lesions [18].

Information on the nodal status remains a crucial component of clinical staging and decision-making. In a review of four studies by Schimmer et al. [19] integrated ^18^F-FDG PET/CT detected mediastinal lymph node metastases with a sensitivity of between 69 and 94%, a specificity of between 86 and 94%, PPV of between 49 and 93%, and NPV were of between 95 and 99%. In our study ^18^F-FDG PET/CT predicted mediastinal nodal metastases with a sensitivity of 57%, a specificity of 95%, PPV of 88% and NPV of 76%. Differences in reported sensitivities may be due to heterogeneity between the different study populations (in terms of histology, tumor size, metabolic rate) but all studies demonstrate – to varying degrees – that lack of glucose uptake in the ^18^F-FDG PET/CT does not guarantee that no nodal metastases are present. The particularly low sensitivity of 55% for lymph node station #7 underscores the importance of careful pathological examination here, potentially preoperatively. Interestingly, ^18^F-FDG PET/CT had the highest sensitivity for lymph node stations #10R and #5 with 80 and 83%, respectively. Our results are in accordance with those published by Cerfolio et al. who reported a sensitivity of 50% for station #7, 83% for station #10R, and 100% for station #5 [20]. In summary, ^18^F-FDG PET/CT is another tool in the toolbox of staging. It may prevent patients from unnecessary lung resection by identifying mediastinal nodal or extrathoracic metastases not detectable with CT alone. On the other hand, however, false positive results can lead to patients with potentially resectable tumors being denied curative surgery. Today ^18^F-FDG PET/CT and brain MRI are considered the standards of preoperative staging to rule out extrapulmonary metastatic disease (M1). Two extensive meta-analyses on the role of ^18^F-FDG PET/CT in detecting distant metastases have reported sensitivities of 77–93% and specificities of 95–96% [21, 22]. Interestingly, we found that ^18^F-FDG PET/CT predicted distant metastases with a sensitivity of 100%, a specificity of 98%, and a positive predictive value of 58%.

We found that PET/CT upstaged T descriptor in 15.7% of cases and downstaged it in 9.8%; the N descriptor was upstaged in 18.8% and downstaged in 7.5%, respectively. In other words, PET/CT was more than twice as likely to overestimate tumor stage as to underestimate it. For this reason, we argue for the liberal use of careful histological investigation, particularly in borderline cases, before deeming patients non-operable. In cases of suspected nodal metastases, along with potential T3 and T4 involvement of adjacent structures, intraoperative evaluation may still be justified.

One strength of our study was the long-term follow-up period and the strict prospective protocol of a relatively large patient cohort. All patients included in the survival analysis underwent curative surgical resection, and pathological findings were used to judge the accuracy of ^18^F-FDG PET/CT -staging. The long observation period, however, also presents a limitation in that it includes a time in which various new systemic treatments – especially for patients with recurrent disease –became widespread in clinical practice. We have no good method for accounting for the possible effects of these new treatments on long-term survival. Another limitation of the study is that the sensitivity and specificity of ^18^F-FDG PET/CT may be influenced by the different histologies of non-small cell lung cancer. Furthermore, we did not analyze additional SUV parameters such as metabolic tumor volume or total lesion glycolysis, which may be sources of further useful information about the tumor characteristics.

## Conclusions

This study underscores the role of integrated ^18^F-FDG PET/CT as a valuable instrument in preoperative staging. We have demonstrated, however, that clinical stage based on ^18^F-FDG PET/CT often differs from the pathological stage, providing evidence of the need for further invasive staging in many cases. In lymph node station #7 the reliability of ^18^F-FDG PET/CT is uncertain, suggesting the need for EBUS bronchoscopy or even perhaps more invasive diagnostic techniques such as mediastinscopy. In contrast to some authors [23], we could not confirm that preoperative SUV values independently predict overall survival. Thus, we advise against basing preoperative treatment decisions on SUVmax or SUVmean alone, without taking all relevant tumor and clinical information into account.

## Supplementary Material

Supplementary Material

## References

[1] Sung H, Ferlay J, Siegel RL, Laversanne M, Soerjomataram I, Jemal A (2021). Global cancer statistics 2020: GLOBOCAN estimates of incidence and mortality Worldwide for 36 cancers in 185 Countries. CA Cancer J Clin.

[2] Mountain CF (1997). Revisions in the international system for staging lung cancer. Chest.

[3] Goldstraw P, Chansky K, Crowley J, Rami-Porta R, Asamura H, Eberhardt WEE (2016). The IASLC lung cancer staging project: proposals for revision of the TNM stage groupings in the forthcoming (eighth) edition of the TNM Classification for lung cancer. J Thorac Oncol.

[4] Dienemann H, Hoffmann H, Koebe HG (1998). Technik und rationale der lymphknotendissektion bei bronchialcarcinom. Chirurg.

[5] Kaplan EL, Meier P (1958). Nonparametric estimation from incomplete observations. J Am Stat Assoc.

[6] Downey RJ, Akhurst T, Gonen M, Park B, Rusch V (2007). Fluorine-18 fluorodeoxyglucose positron emission tomographic maximal standardized uptake value predicts survival independent of clinical but not pathologic TNM staging of resected non-small cell lung cancer. J Thorac Cardiovasc Surg.

[7] Sasaki R, Komaki R, Macapinlac H, Erasmus J, Allen P, Forster K (2005). [18F]fluorodeoxyglucose uptake by positron emission tomography predicts outcome of non-small-cell lung cancer. J Clin Oncol.

[8] Davies A, Tan C, Paschalides C, Barrington SF, O’Doherty M, Utley M (2007). FDG-PET maximum standardised uptake value is associated with variation in survival: analysis of 498 lung cancer patients. Lung Cancer.

[9] Ventura L, Scarlattei M, Gnetti L, Silini EM, Rossi M, Tiseo M (2020). Prognostic value of [18F]FDG PET/CT parameters in surgically resected primary lung adenocarcinoma: a single-center experience. Tumori.

[10] Vesselle H, Freeman JD, Wiens L, Stern J, Nguyen HQ, Hawes SE (2007). Fluorodeoxyglucose uptake of primary non-small cell lung cancer at positron emission tomography: new contrary data on prognostic role. Clin Cancer Res.

[11] Higashi K, Ueda Y, Arisaka Y, Sakuma T, Nambu Y, Oguchi M (2002). 18F-FDG uptake as a biologic prognostic factor for recurrence in patients with surgically resected non-small cell lung cancer. J Nucl Med.

[12] Dhital K, Saunders CAB, Seed PT, O’Doherty MJ, Dussek J (2000). [(18)F]Fluorodeoxyglucose positron emission tomography and its prognostic value in lung cancer. Eur J Cardio Thorac Surg.

[13] Cerfolio RJ, Bryant AS, Ohja B, Bartolucci AA (2005). The maximum standardized uptake values on positron emission tomography of a non-small cell lung cancer predict stage, recurrence, and survival. J Thorac Cardiovasc Surg.

[14] Agarwal M, Brahmanday G, Bajaj SK, Ravikrishnan KP, Wong CYO (2010). Revisiting the prognostic value of preoperative (18)F-fluoro-2-deoxyglucose ( (18)F-FDG) positron emission tomography (PET) in early-stage (I & II) non-small cell lung cancers (NSCLC). Eur J Nucl Med Mol Imag.

[15] Ito T, Noguchi Y, Satoh S, Hayashi H, Inayama Y, Kitamura H (1998). Expression of facilitative glucose transporter isoforms in lung carcinomas: its relation to histologic type, differentiation grade, and tumor stage. Mod Pathol.

[16] Cronin P, Dwamena BA, Kelly AM, Carlos RC (2008). Solitary pulmonary nodules: meta- analytic comparison of cross-sectional imaging modalities for diagnosis of malignancy. Radiology.

[17] Kim SK, Allen-Auerbach M, Goldin J, Fueger BJ, Dahlbom M, Brown M (2007). Accuracy of PET/CT in characterization of solitary pulmonary lesions. J Nucl Med.

[18] Patel VK, Naik SK, Naidich DP, Travis WD, Weingarten JA, Lazzaro R (2013). A practical algorithmic approach to the diagnosis and management of solitary pulmonary nodules: part 1: radiologic characteristics and imaging modalities. Chest.

[19] Schimmer C, Neukam K, Elert O (2006). Staging of non-small cell lung cancer: clinical value of positron emission tomography and mediastinoscopy. Interact Cardiovasc Thorac Surg.

[20] Cerfolio RJ, Ojha B, Bryant AS, Raghuveer V, Mountz JM, Bartolucci AA (2004). The accuracy of integrated PET-CT compared with dedicated PET alone for the staging of patients with nonsmall cell lung cancer. Ann Thorac Surg.

[21] Wu Y, Li P, Zhang H, Shi Y, Wu H, Zhang J (2013). Diagnostic value of fluorine 18 fluorodeoxyglucose positron emission tomography/computed tomography for the detection of metastases in non-small-cell lung cancer patients. Int J Cancer.

[22] Li J, Xu W, Kong F, Sun X, Zuo X (2013). Meta-analysis: accuracy of 18FDG PET-CT for distant metastasis staging in lung cancer patients. Surg Oncol.

[23] Liu J, Dong M, Sun X, Li W, Xing L, Yu J (2016). Prognostic value of 18F-FDG PET/CT in surgical non-small cell lung cancer: a meta-analysis. PLoS One.

