# Supplementary material for: Value of 18F FDG-PET/CT parameters on long term follow-up for patients with non-small cell lung cancer
Source: Innov Surg Sci. 2022 Oct 5;7(2):35–43. doi: 10.1515/iss-2022-0009 (PMC9574653; doi:10.1515/iss-2022-0009)
Supplement: Supplementary file 1 — Supplementary Material [file j_iss-2022-0009_suppl.pdf]

Reviewer Assessment

J. Pfannschmidt et al.: Value of 18F FDG-PET/CT parameters on long term follow-up for patients with non-small cell lung cancer

Reviewers' Comments to Original Submission

Reviewer 1: anonymous

Date received: 11-Apr-2022
Reviewer recommendation: **Return to author for minor modifications**
Reviewer overall scoring: **High**

Assessment Form scores: 5 = High/Yes; 3 = Medium/Adequate; 1 = Low

Is the subject area appropriate for the journal	5				
Does the title clearly reflect the paper's content?	5				
Does the abstract clearly reflect the paper's content	5				
Do the keywords clearly reflect the paper's content?	5				
Does the introduction present the problem clearly?	5				
Are the results/ conclusions justified?	5				
How comprehensive and up-to-date is the subject matter presented?	5				
How adequate is the data presentation?	5				
Are units and terminology used correctly?	5				
Is the number of cases adequate?			3		
Are the experimental methods/ clinical studies adequate?		4			
Is the length appropriate in relation to the content?		4			
Does the reader get new insights from the article?		4			
Please rate the practical significance.	5				
Please rate the accuracy of methods.	5				
Please rate the statistical evaluation and quality control.	5				
Please rate the appropriateness of the figures and tables.	5				
Please rate the appropriateness of the references.	5				
Please evaluate the writing style and use of language.		4			
Please judge the overall scientific quality of the manuscript.		4			
Are the methods used worthy of reproduction in greater deal?	Yes				
Would you be willing to review a revision of this manuscript?	Yes				

Comments to author: Dear authors,
the paper describes the prospective study of a cohort of patients, which had a preoperative PET-CT. The prognostic value to predict survival was tested.
The design of the study is of clinical interest and complete.
The methods used were adequate and the text is well written.
The discussion reflects the results of the study and the literature is complete.
The study confirms the deep impact of lymph node metastases on the survival of the patients.
The prognostic value of SUV in PET-CT may be most relevant in stage I lung cancer, since the discrimination between malign and benign coin lesions is clinically important and the indication for a surgical intervention is based on the findings of an PET-CT repeatedly. Your study relativises this clinical procedure. Low SUV lesions carry the same oncological risk like high SUV tumors.
I did not found the sensitivity of PET-CT or SUV of the primary tumor or the number of lung cancers, which where negative.
The study included a wide range of stages of lung cancer and the number of Stage I tumors is rather low (37,6% / 96 pts.). Therefore the power of the study to predict the prognostic value of PET-CT in stage I lung cancer patients may be limited.
Please discuss this clinically relevant aspect..

Reviewer 2: Hecker, Erich

Date received: 22-May-2022
Reviewer recommendation: **Return to author for minor modifications**
Reviewer overall scoring: **High**

Assessment Form scores: 5 = High/Yes; 3 = Medium/Adequate; 1 = Low

Is the subject area appropriate for the journal	5				
Does the title clearly reflect the paper's content?	5				
Does the abstract clearly reflect the paper's content	5				
Do the keywords clearly reflect the paper's content?	5				
Does the introduction present the problem clearly?	5				
Are the results/ conclusions justified?	5				
How comprehensive and up-to-date is the subject matter presented?		4			
How adequate is the data presentation?		4			
Are units and terminology used correctly?	5				
Is the number of cases adequate?	5				
Are the experimental methods/ clinical studies adequate?	5				
Is the length appropriate in relation to the content?	5				
Does the reader get new insights from the article?			3		
Please rate the practical significance.		4			
Please rate the accuracy of methods.		4			
Please rate the statistical evaluation and quality control.	5				
Please rate the appropriateness of the figures and tables.	5				
Please rate the appropriateness of the references.		4			
Please evaluate the writing style and use of language.		4			
Please judge the overall scientific quality of the manuscript.		4			
Are the methods used worthy of reproduction in greater deal?	Yes				

Would you be willing to review a revision of this manuscript?	Yes

Comments to author: Dear authors,
thank you for the opportunity to review the manuscript "Value of 18F FDG-PET/CT parameters on long term follow-up for patients with non-small cell lung cancer" for Innovative Surgical Sciences.
You spend a lot of effort in preparation of this manuscript and are according to the STROBE checklist.

I got few annotations:
- You adhere to a homogenous staging algorithm for all patients, according to the guidelines.
- Was the preoperative cerebral staging waived independently of T-stage?
- The results of lymph node staging by PET/CT are very detailed in Table 4.
- Please add the p-value to the Kaplan-Meier-curves.
- Did all patients with positive lymphnodes in PET/CT receive invasive staging?
- Did any patients receive induction therapy?
- Median duration of 16 days between PET/CT scan and curative resection is very fast.
- The results are clearly presented and the discussion is comprehensible.
- The figures are very well and give good illustration., please correct.

Authors' Response to Reviewer Comments

Date received: 11-Jun-2022

Response to reviewer 1

The reviewer asked about the sensitivity of PET-CT for detecting the primary tumor and the number of histologically proven lung cancers, which were PET-negative. This has been added in the results section:
"The sensitivity for 18F-FDG PET/CT scans (SUVmax ≥2.5) for detecting primary lung cancer was 96.8% (8 of 255 patients were PET negative); for pT1 tumors the sensitivity was 90.9% (7 of 77 patients were PET negative)."
We acknowledge that given the small sample size, this study may be underpowered to detect potential prognosticators in pT1 patients. According to the suggestions of the reviewer we added in the discussion section:
"The management of T1 tumors (coin like solitary pulmonary nodules) in the routine staging process with 18F-FDG PET/CT is evolving. The reported sensitivity of 90.9% in our study for T1 tumors has been also shown in other publications, with reported sensitivities of up to 95% and 97% (Cronin P, Dwamena BA, Kelly AM, Carlos RC. et al.). However, the threshold of 8—10mm in tumor size for PET examinations was set in international guidelines to underline the significant risk of false-negative findings for small coin lesions (Patel VK, Naik SK, Naidich DP, Travis WD et al.). To eliminate one of the main sources of false-negative and false-positive findings, PET should not be considered reliable for evaluating ground glass opacity lesions.

Response to reviewer 2

The review asked whether preoperative cerebral staging was waived, regardless of T-stage. We added for clarity in the methods section: "In patients with advanced stage lung cancer an MRI was routinely performed. However, in patients with stage I and II disease, cerebral staging relied mainly on the contrast-enhanced 18F-FDG PET/CT imaging protocols."

The reviewer suggested to providing the p-values for the Kaplan-Meier curves. We added the p-values accordingly to the figure legends.

The reviewer asked whether all patients with positive lymph nodes in PET/CT received invasive staging. Although all patients had bronchoscopy prior surgery, invasive lymph node staging by EBUS-bronchoscopy or mediastinoscopy was primarily performed in patients with suspected mediastinal lymphadenopathy. We added this information in the methods section.

None of the patients had induction therapy because this would have altered the treatment protocol. We added this information in the methods section.

Reviewers' Comments to the Revised Manuscript

Reviewer 1: anonymous

Date received: 12-Jul-2022
Reviewer recommendation: **Accept in present form**
Reviewer overall scoring: **High**

Assessment Form scores: 5 = High/Yes; 3 = Medium/Adequate; 1 = Low

Is the subject area appropriate for the journal		4			
Does the title clearly reflect the paper's content?	5				
Does the abstract clearly reflect the paper's content	5				
Do the keywords clearly reflect the paper's content?	5				
Does the introduction present the problem clearly?	5				
Are the results/ conclusions justified?	5				
How comprehensive and up-to-date is the subject matter presented?	5				
How adequate is the data presentation?		4			
Are units and terminology used correctly?		4			
Is the number of cases adequate?		4			
Are the experimental methods/ clinical studies adequate?		4			
Is the length appropriate in relation to the content?		4			
Does the reader get new insights from the article?		4			
Please rate the practical significance.	5				
Please rate the accuracy of methods.		4			
Please rate the statistical evaluation and quality control.		4			
Please rate the appropriateness of the figures and tables.					
Please rate the appropriateness of the references.		4			
Please evaluate the writing style and use of language.		4			
Please judge the overall scientific quality of the manuscript.		4			
Are the methods used worthy of reproduction in greater deal?	No				
Would you be willing to review a revision of this manuscript?	Yes				

Comments to author: No further comments.

Comments by the Editor-in-Chief to Revised Submission

All reviewer comments were addressed adequately and the manuscript should be published in its present stage.
